# Genetic determinants of sporadic breast cancer in Sri Lankan women

**DOI:** 10.1186/s12885-018-4112-4

**Published:** 2018-02-13

**Authors:** Nirmala Dushyanthi Sirisena, Adebowale Adeyemo, Anchala I. Kuruppu, Nilaksha Neththikumara, Nilakshi Samaranayake, Vajira H. W. Dissanayake

**Affiliations:** 1 Human Genetics Unit, Faculty of Medicine, University of Colombo, Kynsey Road, Colombo 8, Sri Lanka; 2 Center for Research on Genomics and Global Health, National Human Genome Research Institute, Bethesda, MD USA; 3 Department of Parasitology, Faculty of Medicine, University of Colombo, Colombo, Sri Lanka

**Keywords:** Haplotypes, Postmenopausal women, Sporadic breast cancer, Susceptibility

## Abstract

**Background:**

While a range of common genetic variants have been identified to be associated with risk of sporadic breast cancer in several Western studies, little is known about their role in South Asian populations. Our objective was to examine the association between common genetic variants in breast cancer related genes and risk of breast cancer in a cohort of Sri Lankan women.

**Methods:**

A case-control study of 350 postmenopausal women with breast cancer and 350 healthy postmenopausal women was conducted. Genotyping using the iPLEX GOLD assay was done for 56 haplotype-tagging single nucleotide polymorphisms (SNPs) in 36 breast cancer related genes. Testing for association was done using an additive genetic model. Odds ratios and 95% confidence intervals were calculated using adjusted logistic regression models.

**Results:**

Four SNPs [rs3218550 (*XRCC2*), rs6917 (*PHB*), rs1801516 *(ATM)*, and rs13689 *(CDH1)*] were significantly associated with risk of breast cancer. The rs3218550 T allele and rs6917 A allele increased breast cancer risk by 1.5-fold and 1.4-fold, respectively. The CTC haplotype defined by the SNPs rs3218552|rs3218550|rs3218536 on chromosome 7 (*P* = 0.0088) and the CA haplotype defined by the SNPs rs1049620|rs6917 on chromosome 17 (*P* = 0.0067) were significantly associated with increased risk of breast cancer. The rs1801516 A allele and the rs13689 C allele decreased breast cancer risk by 0.6-fold and 0.7-fold, respectively.

**Conclusions:**

These findings suggest that common genetic polymorphisms in the *XRCC2*, *PHB, CDH1* and *ATM* genes are associated with risk of breast cancer among Sri Lankan postmenopausal women. The exact biological mechanisms of how these variants regulate overall breast cancer risk need further evaluation using functional studies.

**Electronic supplementary material:**

The online version of this article (10.1186/s12885-018-4112-4) contains supplementary material, which is available to authorized users.

## Background

Breast cancer is a multifactorial disease that results from the association between various genetic, environmental, hormonal and lifestyle factors [1]. It is the commonest cancer in women and the major cause of cancer mortality among women all over the world [2]. In Sri Lanka, breast cancer accounts for approximately 23% of all cancers in females. It is also the main cancer contributing to 12% of all cancers among Sri Lankans [3]. Compared to Western countries, survival after breast cancer is generally poorer in Sri Lankan women due to delayed diagnosis and inadequate standard management protocols [3].

The development of breast cancer is known to involve a multistep process associated with numerous genetic alterations. It is hypothesized that a multitude of low-to-moderate penetrance or modifier genetic alleles that are polymorphic in the population, each conferring a small increase in the overall risk (ranging from just over 1.0 to 2.0 fold), and various environmental factors may contribute to the underlying risk for sporadic breast cancer [4]. Recent genetic studies, especially genome-wide association studies (GWAS), in European, African-American and East-Asian populations have revealed numerous common genetic variants associated with susceptibility to breast cancer [5, 6]. Notably, most studies of sporadic breast cancer genetic risk have been done in populations of European ancestry [7–11]. Recently, the International Breast Cancer Association Consortium (BCAC) carried out a large GWAS involving participants from Europe, North America, South-East Asia and Australia, and detected 5 single nucleotide polymorphisms (SNPs) that were related to breast cancer. Four were located within haplotype blocks containing genes: rs2981582 in intron 2 of the *FGFR2* gene at chromosome 10q; rs889312 near *MAP3K1* at 5q; rs3803662 located between *CASC16* and the LOC643714 gene at 16q; intronic variant rs3817198 located in *LSP1* at 11p and rs13281615 located at 8q24 in a region lacking any annotated genes [10].

However, there is paucity of data regarding the genetic risk factors for breast cancer in South Asian populations. As part of the Asia Breast Cancer Consortium, Long et al. carried out a multi-stage GWAS to identify susceptible genetic loci for breast cancer in more than 28,000 cases and controls involving women of Asian and European American ancestry [7]. Their results provided strong evidence implicating intronic variant rs4784227 on *CASC16* gene as a functional variant causing breast cancer at the 16q12.1 locus in Asian women [OR = 1.25, 95% CI = 1.20–1.31, *P* = 3.2 × 10^− 25^]. They demonstrated the functional significance of this intronic variant using a combination of luciferase reporter gene assays and electrophoretic mobility shift assays. Similarly, Mazhar et al. reported that two intronic variants of *FGFR2* [rs2981582 (*P* = 0.005), rs1219648 (*P* = 9.08e-006)] and a non-coding transcript of *CASC16* [rs3803662 (*P* = 0.012)] were related to sporadic breast cancer in Pakistani women [12].

The few studies on genetic susceptibility for breast cancer in the Sri Lankan population have focused mainly on the *BRCA1* and *BRCA2* genes [13–15]. There are still no published studies that have comprehensively investigated the association between genetic polymorphisms in breast cancer related genes and risk of sporadic breast cancer in Sri Lankan women [13–15]. Having a knowledge of such risk variants would be useful in predicting the risk of breast cancer in the local population and facilitating risk reduction and surveillance strategies.

In the current study, we carry out a comprehensive evaluation of genetic polymorphisms associated with sporadic breast cancer in Sri Lankan women. Using a case-control approach, we conducted association testing on 700 women using single variant and haplotype [16] association testing strategies on 56 SNPs in 36 breast cancer related genes.

## Methods

### Study design and study population

The study used a case-control design. An already existing EDTA blood resource which has been collected with the approval of the Ethics Review Committee, Faculty of Medicine, University of Colombo, for this sort of genetic studies was used for this study. The study participants had provided written informed consent for their samples to be used for future genetic studies related to breast cancer with ethics approval. The samples were collected from a Sri Lankan cohort of 350 unrelated postmenopausal women with histologically confirmed invasive breast cancer (cases), and 350 unrelated postmenopausal women who have never been diagnosed to have any malignancy (controls). All study participants were recruited in Colombo, Sri Lanka between March 2010 and October 2011. The cases were recruited from patients detected with sporadic breast cancer, at the time of reviewing their histology report following mastectomy, and prior to commencement of chemotherapy or radiotherapy. The controls had been recruited from the general population by open advertisement. Both the control group and cases were of the same ethnicity (Sinhalese). Any participant with a family history of any type of cancer in their first- and second-degree relatives was excluded. This was done to exclude individuals with hereditary cancer syndromes. In addition, any control who had previously been diagnosed with any type of malignancy was also excluded. The epidemiological data of the cases and controls were retrieved from an electronic database kept at the Human Genetics Unit, Faculty of Medicine, University of Colombo.

The sample size of 350 cases and 350 controls can detect a two-fold increased risk of breast cancer for any genetic marker present with a minor allele frequency of at least 10% in the population with 80% power, at a significance level of *P*-value < 0.05 [17].

### Selection of SNPs for genotyping

The candidate genes associated with sporadic breast cancer which encode either for transcription factors, proto-oncogenes, tumour suppressors, proteins involved in signal transduction pathways, cell cycle checkpoint and/or DNA repair pathways that have so far been reported were first identified through survey of published scientific literature. The first step in selecting the haplotype tagging SNPs involved mining the available data at the Human HapMap database (http://hapmap.ncbi.nlm.nih.gov/) for SNPs in the identified breast cancer candidate genes that were polymorphic (minor allele frequencies above 0.05) in the South Asian population: HapMap Gujarati Indians in Houston, Texas (GIH). Genotype data from this population group was used for haplotype tagging because at the time the study was designed, this was the only South Asian population group in the International HapMap project or other similar projects with dense genotypes. HapMap Genome Browser release #27 Phase 1, 2 and 3 merged genotype and frequency data was selected and linkage disequilibrium (LD) analysis was carried out using the default algorithm. Selection of non-synonymous SNPs in the coding regions, and SNPs in the 5′-untranslated regions (5’UTR) and the 3′-untranslated regions (3’UTR) of the candidate genes was performed using the SNP nexus software tool (http://snp-nexus.org/). Coding synonymous and intronic SNPs were excluded.

The tagger algorithm implemented in Haploview 4.2 (http://www.broad.mit.edu/mpg/haploview/) was used for the selection of haplotype tagging SNPs. Tag SNP selection was carried out using the pairwise tagging method using a D’ threshold of 0.8. Further refining and prioritisation of the haplotype tagging SNPs was done using various bioinformatics software tools with regard to the validation status of the SNP (dbSNP database; http://www.ncbi.nlm.nih.gov/SNP/), presence within an evolutionary conserved region (MutationTaster; http://www.mutationtaster.org/), and putative functional significance of the variants (F-SNP; http://compbio.cs.queensu.ca/F-SNP/). Altogether, a total of 58 SNPs from 36 breast cancer related candidate genes were selected for genotyping (Additional file 1: Table S1).

### Genotyping and quality control

DNA was extracted from all samples using the Promega Wizard® Genomic DNA purification kit according to the manufacturers’ protocol. The DNA samples were quantified using the Promega QuantiFluor® dsDNA System and normalized to 10.0 ng/μl. All the samples were genotyped for the selected tagged SNPs in the breast cancer candidate genes using the Agena Bioscience Mass-Array technology on a Compact Spectrometer, iPLEX GOLD chemistry (Australian Genome Research Facility, Gehrmann Laboratories, University of Queensland). MassArray Designer software was used for designing primers flanking the gene region containing the SNPs. Altogether, 57 SNPs were successfully genotyped, and the average SNP call rate was 99.87% in both cases and controls. Among them, SNP rs1047111 deviated from Hardy-Weinberg equilibrium test with a *P*-value < 0.05 and was excluded from analysis. A total of 697 subjects, comprising of 349 cases and 348 controls passed filters and quality control.

### Statistical analysis

Differences in demographic features between cases and controls were compared by Chi-squared (χ2) test for categorical variables and Student’s t-test for continuous variables. For each SNP, the allele frequencies and genotype frequencies were compared, and Hardy-Weinberg equilibrium was analysed using χ2 test [18]. In order to determine the association between each SNP and the overall breast cancer risk, the odds ratio (OR) and 95% confidence interval (CI) were analysed using multiple logistic regression analysis with adjustments for age based on the log-additive genetic model using PLINK software version 1.07 (http://pngu.mgh.harvard.edu/purcell/plink/) [19]. A *P*-value < 0.05 was considered statistically significant. Estimation of haplotype frequencies and haplotype association tests for haplotypes with frequencies at least 5% were carried out using PLINK software version 1.07.

## Results

The mean age at diagnosis for the breast cancer cases was 60 (SD 7) years and mean age at recruitment for the controls was 74 (SD 6) years (*P* = 0.001). No significant difference in the age at menarche was found between the cases and controls (*P* = 0.81). Cases were more likely to be older at first pregnancy (*P* = 0.007), and have fewer childbirths (*P* = 0.001), late age at menopause (*P* = 0.003), and higher body mass index (*P* = 0.001) than controls (Table 1).

**Table 1:** Clinical and demographic features of the study participants

Variable	Cases (*n* = 349)	Controls (*n* = 348)	*P*-value
^a^Mean age (years) ^b^[SD]	60 [7]	74 [6]	0.001
Mean age at menarche (years) [SD]	14 [2]	14 [2]	0.81
Mean age at first pregnancy (years) [SD]	26 [7]	25 [6]	0.007
Mean number of children [SD]	3 [2]	4 [3]	0.001
Mean age at menopause (years) [SD]	48 [4]	47 [6]	0.003
Mean body mass index (kg/m^2^) [SD]	25 [4]	23 [4]	0.001

^a^[Cases – age at diagnosis of cancer; Controls – age at recruitment]

^b^Standard deviation

Four SNPs showed significant association with breast cancer:**rs3218550**, NC_000007.14:g.152646870C > T,NG_027988.1:g.34296G > A,NM_005431.1:c.*1772G > A [X-ray repair cross-complementing gene-2 *(XRCC2)/7q36.1*]; **rs6917**,NC_000017.11:g.49404181G > A,NG_023046.1:g.15700C > T,NM_001281496.1:c.*811C > T [prohibitin-1 gene (*PHB)/17q21.33*]; **rs1801516**,NC_000011.9:g.108175462G > A,NG_009830.1:g.86904G > A,NM_000051.3:c. *5557G > A,NP_000042.3:p.Asp1853Asn [ataxia telangiectasia-mutated (*ATM)/11q22.3*]; and **rs13689**,NC_000016.9:g.68868522 T > C,NG_008021.1:g.102328 T > C,NM_001317184.1:c.*1120 T > C [E-cadherin gene (*CDH1)/16q22.1*].

The SNPs rs3218550 [*P* = 0.009837, OR = 1.525, 95% CI = 1.107–2.101] and rs6917 (*P* = 0.006227, OR = 1.41, 95% CI = 1.102–1.803) were found to be significantly associated with breast cancer. Two other SNPs: rs1801516 (*P* = 0.01629, OR = 0.5948, 95% CI = 0.3894–0.9088) and (*P* = 0.03437, OR = 0.6965, 95% CI = 0.4982–0.9737) also had significant association with breast cancer (Table 2). The remaining 52 SNPs did not show any evidence of association with breast cancer (Additional file 2: Table S2). Haplotype association tests showed that the CTC haplotype defined by the SNPs rs3218552|rs3218550|rs3218536 on the *XRCC2* gene on chromosome 7 (*P* = 0.008762) and the CA haplotype defined by the SNPs rs1049620|rs6917 on the *PHB* gene on chromosome 17 (*P* = 0.006718) were significantly associated with increased risk of breast cancer (Table 3).

**Table 2:** Single nucleotide polymorphisms significantly associated with breast cancer risk

^a^Chr	Gene	^b^SNP	Location	Variant allele	Ancestral allele	Variant allele frequency [Cases]	Variant allele frequency [Controls]	Odds ratio	95% Confidence Interval	*P*-value
7	*XRCC2*	rs3218550	3’UTR	T	C	0.1517	0.1049	1.525	1.107–2.101	0.0098
11	*ATM*	rs1801516	Exonic	A	G	0.0544	0.0876	0.595	0.389–0.909	0.0163
16	*CDH1*	rs13689	3’UTR	C	T	0.0988	0.1351	0.697	0.498–0.974	0.0344
17	*PHB*	rs6917	3’UTR	A	G	0.2937	0.2298	1.41	1.102–1.803	0.0062

^a^Chromosome

^b^Single nucleotide polymorphism

**Table 3:** Haplotypes associated with breast cancer risk

Gene	Single nucleotide polymorphisms	Haplotype	Frequency [Cases]	Frequency [Controls]	Chi square	Degree of freedom	*P*-value
*XRCC2*	rs3218552|rs3218550|rs3218536	CTC	0.1519	0.1049	6.871	1	0.0088
*PHB*	rs1049620|rs6917	CA	0.2937	0.2298	7.347	1	0.0067

## Discussion

A case–control study was conducted to investigate the association of SNPs in the transcribed and regulatory regions of breast cancer related genes and the susceptibility to sporadic breast cancer among Sri Lankan postmenopausal women. This is the first study to investigate the association of selected genetic polymorphisms in breast cancer related genes (apart from *BRCA1* and *BRCA2*) and the susceptibility to sporadic breast cancer in the Sri Lankan population. All the cases in this cohort were patients with sporadic breast cancer. Although they were not sequenced for *BRCA1* and *BRCA2* mutations, their advanced age and the lack of a family history of cancer make them unlikely to be mutation carriers. Among 56 tested SNPs, four (rs3218550, rs6917, rs1801516, and rs13689) were significantly associated with risk of breast cancer. The findings of this study provide evidence of genetic susceptibility to overall sporadic breast cancer risk in the Sri Lankan population for the first time.

### X-ray repair cross-complementing gene-2 *(XRCC2)*

In this study, the T allele in the SNP rs3218550 (*XRCC2*) and the A allele in SNP rs6917 (*PHB*) were susceptible alleles for sporadic breast cancer. The SNP rs3218550 at 7q36.1 resides in the 3’UTR of the *XRCC2* gene. A 1.5-fold increase in the risk for breast cancer in women with the T allele compared to those with the C allele was observed. The frequency of the CTC haplotype defined by the SNPs rs3218552|rs3218550|rs3218536 on the *XRCC2* gene had a significant association with increased risk of breast cancer. The observed effect for the *XRCC2* CTC haplotype was stronger than for the rs3218550 polymorphism acting alone. In contrast to our findings, the SNP rs3218536 (also known as Arg188His in exon 3 of the *XRCC2* gene), acting individually was reported to be associated with a slightly protective effect for breast cancer in a study of 1100 Cypriot women [OR = 0.79; 95% CI = 0.62–1.00; *P* = 0.05] [6]. The putative function of this *XRCC2* variant in susceptibility to breast cancer is somewhat unclear. Several studies have shown a correlation with a higher risk of breast cancer while others have not [6, 20–22]. A couple of studies have observed a minor protective effect for breast cancer in individuals with the rs3218536 variant allele [6, 23]. Some other studies reported no significant association in Australian and Korean populations [24, 25]. A slightly increased breast cancer risk in variant allele homozygotes was reported by Kuschel et al. in a Caucasian cohort [*P* = 0.07; OR = 2.6; 95% CI = 1.0–6.7] [26]. However, an increased risk was not found in heterozygotes [OR = 0.9; 95% CI = 0.8–1.1]. A case-control study conducted in a Portuguese population showed decreased breast cancer risk among women that had never breast fed who were heterozygotes for the rs3218536 variant allele [adjusted OR = 0.45; 95% CI = 0.22–0.92); *P* = 0.03] [20]. Similarly, another study conducted in the Polish population reported a decreased risk for breast cancer in homozygotes for the 188His allele [27]. No significant correlation with risk of breast cancer was detected for this SNP in two meta-analyses which included more than 30,000 cases and controls. The authors however mentioned that the possibility of SNP–SNP or SNP–environment interactions resulting in increased breast cancer risk cannot be excluded completely [21, 28]. The conflicting evidence for these associations may be due to ethnic and geographic factors resulting from different carcinogenic exposures of studied populations.

*XRCC2* plays a vital function in the homologous recombination repair (HRR) pathway of double-strand breaks (DSB) in DNA, mainly in the late S and G2 phases, which repairs fragmentations, deletions, translocations in chromosomes, and maintains their stability [29]. It also forms a heterodimer complex with other members of the RAD51 protein family such as RAD51B, RAD51C, RAD51D, XRCC3, BRCA1, and BRCA2. This heterodimer is needed for the localization of RAD51, which plays a key role in mediating HRR at DSB sites [30, 31]. It is postulated that disruption of DSB repair contributes to carcinogenesis through the accumulation of genetic errors and genetic instability [31]. Previous studies have reported that SNPs in the DNA repair pathway genes may exert an effect on breast cancer susceptibility by acting as low penetrance alleles [6]. Even though the *XRCC2* A variant allele of rs3218536 has been shown by cell complementation assays to somewhat augment sensitivity to damage [32], there are no published studies which suggest a relationship between patients with the variant allele of rs3218550 and breast cancer. The precise functional role of rs3218550 in the XRCC2 protein is unknown [33]. We hypothesized that the altered HRR capacity produced by the variant allele of rs3218550 in the 3’UTR of the *XRCC2* gene may influence an individual’s susceptibility to sporadic breast cancer. However, there might be other biological pathways involved, and further functional studies are warranted to elucidate the precise role of this SNP in breast cancer.

### Prohibitin-1 gene (*PHB*)

Our study showed an association between SNP rs6917 (*PHB*) and sporadic breast cancer risk. We observed a 1.4-fold increase in breast cancer risk in women with the A allele compared to those with the G allele. The frequency of the CA haplotype defined by the SNPs rs1049620|rs6917 on the *PHB* gene was significantly associated with increased risk of breast cancer. It is reported that this gene resides in a location that frequently encounters loss of heterozygosity in both hereditary and sporadic breast and ovarian cancers [34]. It has been linked with the regulation of a number of cellular processes, such as cell proliferation and growth, apoptosis, cell signalling, gene transcription, mitochondrial function and control of the oestrogen and androgen receptors [35]. The inactivation of these processes contribute to the pathogenesis of human cancer [35, 36]. Upon phosphorylation, PHB acts as a key mediator in the interaction of Ras with Raf, which results in the upregulation of the MEK-ERK and PI3K signalling pathways, leading to effects on cell adhesion and migration that facilitate cancer progression [37]. The wild-type allele of rs6917, acting individually is known to reduce cell mobility and inhibit cell cycle progression and tumour growth [35]. In addition, the 3’UTR of the *PHB* gene is known to encode a trans-acting regulatory RNA molecule with tumour suppressive effects that impedes proliferation of cells between the G1 and S phases of the cell cycle in both normal epithelial and breast cancer cell lines [38]. The cytosine to thymine transition at position 1630 in the 3’UTR of the rs6917 SNP produces a variant which promotes tumorigenesis through loss of anti-proliferative activity and reduced cell motility [34]. Even though the relationship between rs6917 G/A variant and breast cancer susceptibility has been examined in previous studies; the findings are rather inconsistent [35]. A case-control study conducted in the Polish population reported a significant association between the rs6917 variant allele homozygous genotype and medullary breast cancer [OR = 4.0, 95% CI = 1.1–14.0] [39]. Another study among North American women showed that the variant allele is associated with susceptibility to breast cancer in those aged 50 years and below who had a pedigree with a minimum of one affected first-degree relative [40]. However, case-control studies in Turkish and Australian women showed no association with breast cancer [41, 42]. Its functional effect therefore needs further investigation to clearly identify its relationship with breast cancer in the Sri Lankan population.

### E-cadherin gene (*CDH1*)

The C allele of rs13689 located at 16q22.1 on the 3’UTR of *CDH1*gene was a protective factor for breast cancer in our study (0.7-fold reduction in risk). The *CDH1* gene is mainly involved in cell signalling, maintaining cellular differentiation and intercellular adhesion. A reduced expression of this gene is known to lead to cellular proliferation, invasion, and cancer progression [43]. A systematic evaluation of the common genetic variations in the *CDH1* gene was carried out in a population-based study involving Chinese women [43]. Overall, a correlation with breast cancer risk was not identified among 2083 cases and 2152 controls. However, the authors concluded that among postmenopausal women, the SNP rs13689 was consistently associated with a 1.7 fold increased risk in recessive models [43]. Similarly, another haplotype-based study conducted to investigate the association of *CDH1* with susceptibility to breast cancer in the Chinese Han population, reported that rs13689 was associated with increased risk of breast cancer and poor survival [1]. It is necessary to undertake additional studies to determine the relationship between rs13689 and breast cancer. The *CDH1* gene encodes for the calcium ion-dependent cell adhesion molecule E-cadherin, that is known to function in epithelial integrity and carcinogenesis. It is postulated that reduced expression of E-cadherin may trigger cancer invasion and metastasis [1]. Furthermore, the 3’UTR of genes is essential not only for the stability of mRNA and its localization, but it may also provide the binding site for miRNA. It is reported that common genetic variants in the 3’UTR of a number of genes have been linked to several diseases through their effects on miRNA regulated gene/protein expression [1]. The C allele of SNP rs13689 may exert a protective effect on breast cancer susceptibility through one of these mechanisms and warrants further investigation through functional studies.

### Ataxia telangiectasia-mutated gene (*ATM*)

The A allele of rs1801516, located at 11q22.3 in the coding region of the *ATM* gene showed a reduction in the risk of breast cancer by 0.6-fold. ATM is a serine-threonine kinase that is known to be associated with risk of breast cancer through its regulatory effects on the cellular response to DNA DSB [44]. In addition, active ATM is known to exert antineoplastic effects through the activation of checkpoints in the cell cycle, initiation of apoptosis, and accumulation of p53 [44]. However, several studies have reported conflicting results on the relationship between *ATM* genetic variants and breast cancer susceptibility [42–45]. Mehdipour et al. reported that a common SNP (rs1801516) in exon 39 of the *ATM* gene (5557G > A, D1853N) may serve to increase the risk for breast cancer, mainly in families with cancer predisposition [45]. Shrauder et al. reported a marginal association between this variant and reduced breast cancer risk [OR for the heterozygous genotype = 0.70, 95% CI, 0.52–0.94, *P* = 0.018] and for the homozygous variant, [OR = 0.63, 95% CI, 0.27–1.49, *P* = 0.292] [46]. However, a meta-analysis involving 9 epidemiological studies showed that the *ATM* rs1801516 polymorphism had no association with risk of breast cancer. The authors concluded that this polymorphism acting alone may not influence breast cancer susceptibility [47]. The published data on the role of *ATM* as a marker of genetic susceptibility to breast cancer is rather inconsistent. Concannon et al. reported that carriers of common *ATM* genetic variants had a reduction in the risk of cancer in the contralateral breast which was statistically significant [48]. According to their findings, SNP rs1801673, c.5558A > T, p.Asp1853Val [RR = 0.2; 95% CI, 0.1–0.6] was associated with a substantial reduction in the risk of developing a contralateral breast cancer while rs1801516, 5557G > A, p.Asp1853Val was associated with a mere 0.9 fold reduction in risk [48]. These findings indicate that certain *ATM* alleles may produce an anti-tumour effect, either by modifying the activity of *ATM* through its initial response to DNA damage or as a regulator of p53 [48]. Functional studies of the cellular activity of *ATM* in individuals who carry these variant alleles will aid in further elucidating their anti-tumour properties.

Our study had several strengths, including an adequate sample size from a homogenous ethnicity (100% of study participants were Sinhalese), thus minimizing any bias arising from population stratification. Study limitations include the fact that the analysis did not take into consideration probable differences in lifestyle factors and the selected SNPs may not give as comprehensive a view of genetic variation as sequencing does. It is possible that the other SNPs which showed a null association with breast cancer either do not modify the susceptibility to breast cancer in the Sri Lankan population or their effects are minimal and can be detected only with larger study samples. We plan to address these issues in subsequent studies. A detailed analysis of the phenotypic and clinical characteristics of this cohort in relation to the genotypic results is the subject of another study.

## Conclusions

The findings of this study indicate that common genetic variations in the *XRCC2*, *PHB, CDH1* and *ATM* genes, respectively may influence susceptibility to breast cancer among Sri Lankan postmenopausal women. Considering the vital functional roles of *XRCC2* and *PHB* genes in HRR and cell cycle regulation, the significant differences in genetic susceptibility to breast cancer in the Sri Lankan population observed with the SNPs rs3218550 and rs6917 and the haplotypes defined by them may indicate a true association. However, the exact biological mechanisms by which these polymorphisms regulate overall breast cancer risk needs further evaluation using functional studies. These findings have important implications as data from multiple breast cancer susceptibility alleles may be pooled together to detect women at varying levels of breast cancer risk. This sort of stratification could help guide preventive and screening strategies. Thus, these results may have potential implications in the early detection, prevention and treatment of sporadic breast cancer patients and deserve further investigation through functional assays.

## Additional files

Additional file 1: **Table S1.** List of haplotype-tagging single nucleotide polymorphisms selected for genotyping. Table S1 shows the list of haplotype-tagging single nucleotide polymorphisms which were selected for genotyping including detailed information about the genes, chromosomes, locations and putative functional scores of the genetic variants. (DOCX 23 kb)

Additional file 2: **Table S2** Relationship between the genotyped single nucleotide polymorphisms and breast cancer risk. Table S2 shows the list of haplotype-tagging single nucleotide polymorphisms which were genotyped in the study cohort and their association with breast cancer risk. (DOCX 19 kb)

## Abbreviations

3’UTR: 3′-untranslated region
5’UTR: 5′-untranslated region
*ATM*: Ataxia telangiectasia-mutated
*CDH1*: E-cadherin
CI: Confidence interval
DSB: Double-strand breaks
GWAS: Genome-wide association studies
HRR: Homologous recombination repair
LD: Linkage disequilibrium
OR: Odds ratio
*PHB*: Prohibitin-1
SNPs: Single nucleotide polymorphisms
*XRCC2*: X-ray repair cross-complementing gene-2
χ2: chi-squared

## References

[1] Jia YM, Xie YT, Wang YJ, Han JY, Tian XX, Fang WG (2015). Association of genetic polymorphisms in *cdh1* and *ctnnb1* with breast cancer susceptibility and patients’ prognosis among chinese han women. PLoS One.

[2] Jemal A, Bray F, Center MM, Ferlay J, Ward E, Forman D (2011). Global cancer statistics. CA Cancer J Clin.

[3] Sirisena ND, Dissanayake VHW (2014). Cancer genetics and the surgeon – new frontiers. Sri Lanka J Surg.

[4] Bell DW, Kim SH, Godwin AK, Schiripo TA, Harris PL, Haserlat SM, Wahrer DCR, Haiman CA, Daly MB, Niendorf KB, Smith MM, Sgroi DC, Garber JE, Olopade OI, Le Marchand L, Henderson BE, Altshuler D, Haber DA, Freedman ML (2007). Genetic and functional analysis of *chek2* (*chk2*) variants in multiethnic cohorts. Int J Cancer.

[5] Deng Z, Yang H, Liu Q, Wang Z, Feng T, Ouyang Y, Jin T, Ren H (2016). Identification of novel susceptibility markers for the risk of overall breast cancer as well as subtypes defined by hormone receptor status in the chinese population. J Hum Genet.

[6] Loizidou MA, Michael T, Neuhausen SL, Newbold RF, Marcou Y, Kakouri E, Daniel M, Papadopoulos P, Malas S, Kyriacou K, Hadjisavvas A (2008). Genetic polymorphisms in the dna repair genes *xrcc1*, *xrcc2* and *xrcc3* and risk of breast cancer in cyprus. Breast Cancer Res Treat.

[7] Long J, Cai Q, Shu X-O, Qu S, Li C, Zheng Y, Gu K, Wang W, Xiang YB, Cheng J, Chen K, Zhang L, Zheng H, Shen CY, Huang C-S, Hou MF, Shen H, Hu Z, Wang F, Deming SL, Kelley MC, Shrubsole MJ, Khoo US, Chan KYK, Chan SY, Haiman CA, Henderson BE, Le Marchand L, Iwasaki M, Kasuga Y (2010). Identification of a functional genetic variant at 16q12.1 for breast cancer risk: results from the asia breast cancer consortium. PLoS Genet.

[8] Mavaddat N, Pharoah PD, Michailidou K, Tyrer J, Brook MN, Bolla MK, Wang Q, Dennis J, Dunning AM, Shah M, Luben R, Brown J, Bojesen SE, Nordestgaard BG, Nielsen SF, Flyger H, Czene K, Darabi H, Eriksson M, Peto J, Dos-Santos-Silva I, Dudbridge F, Johnson N, Schmidt MK, Broeks A, Verhoef S, Rutgers EJ, Swerdlow A, Ashworth A, Orr N, et al. Prediction of breast cancer risk based on profiling with common genetic variants. J Natl Cancer Inst. 2015;107 10.1093/jnci/djv036 PMC4754625 25855707

[9] Gaudet MM, Kuchenbaecker KB, Vijai J, Klein RJ, Kirchhoff T, Mcguffog L, Barrowdale D, Dunning AM, Lee A, Dennis J, Healey S, Dicks E, Soucy P, Sinilnikova OM, Pankratz VS, Wang X, Eldridge RC, Tessier DC, Vincent D, Bacot F, Hogervorst FBL, Peock S, Stoppa-Lyonnet D, Peterlongo P, Schmutzler RK, Nathanson KL, Piedmonte M, Singer CF, Thomassen M, KConFab Investigators (2013). Identification of a brca2-specific modifier locus at 6p24 related to breast cancer risk. Plos Genet.

[10] Easton DF, Pooley KA, Dunning AM, Pharoah PDP, Thompson D, Ballinger DG, Struewing JP, Morrison J, Field H, Luben R, Wareham N, Ahmed S, Healey CS, Bowman R, Meyer KB, Haiman CA, Kolonel LK, Henderson BE, Le Marchand L, Brennan P, Sangrajrang S, Gaborieau V, Odefrey F, Shen CY, Wu PE, Wang HC, Eccles D, Evans DG, Peto J, SEARCH Collaborators (2007). Genome-wide association study identifies novel breast cancer susceptibility loci. Nature.

[11] Michailidou K, Hall P, Gonzalez-Neira A, Ghoussaini M, Dennis J, Milne RL, Schmidt MK, Chang-Claude J, Bojesen SE, Bolla MK, Wang Q, Dicks E, Lee A, Turnbull C, Rahman N, Fletcher O, Peto J, Gibson L, Dos Santos Silva I, Nevanlinna H, Muranen TA, Aittomäki K, Blomqvist C, Czene K, Irwanto A, Liu J, Waisfisz Q, Meijers-Heijboer H, Adank M, Breast and Ovarian Cancer Susceptibility Collaboration (2013). Large-scale genotyping identifies 41 new loci associated with breast cancer risk. Nat Genet.

[12] Mazhar A, Jamil F, Bashir Q, Ahmad MS, Masood M, Tanvir I, Rashid N, Waheed A, Afzal MN, Tariq MA (2016). Genetic variants in *fgfr2* and *tnrc9* genes are associated with breast cancer risk in pakistani women. Mol Med Report.

[13] De Silva W, Karunanayake EH, Tennekoon KH, Allen M, Amarasinghe I, Angunawala P, Ziard MH (2008). Novel sequence variants and a high frequency of recurrent polymorphisms in *brca1* gene in sri lankan breast cancer patients and at risk individuals. BMC Cancer.

[14] De Silva S, Tennekoon KH, Karunanayake EH, De Silva W, Amarasinghe I, Angunawela P (2011). Novel sequence variants and common recurrent polymorphisms of *brca2* in sri lankan breast cancer patients and a family with *brca1* mutations. Exp Ther Med.

[15] De Silva S, Tennekoon KH, Karunanayake EH, Amarasinghe I, Angunawela P (2014). Analysis of *brca1* and *brca2* large genomic rearrangements in sri lankan familial breast cancer patients and at risk individuals. BMC Res Notes.

[16] Haiman CA, Stram DO, Pike MC, Kolonel LN, Burtt NP, Altshuler D, Hirschhorn J, Henderson BE (2003). A comprehensive haplotype analysis of *cyp19* and breast cancer risk: the multiethnic cohort. Hum Mol Genet.

[17] Gauderman WJ (2002). Sample size requirements for association studies of gene-gene interaction. Am J Epidemiol.

[18] Wigginton JE, Cutler DJ, Abecasis GR (2005). A note on exact tests of hardy-weinberg equilibrium. Am J Hum Genet.

[19] Purcell S, Neale B, Todd-Brown K, Thomas L, Ferreira M, Bender D, Maller J, Sklar P, De Bakker P, Daly M, Sham P (2007). Plink: a toolset for whole-genome association and population-based linkage analysis. Am J Hum Genet.

[20] Silva SN, Tomar M, Paulo C, Gomes BC, Azevedo AP, Teixeira V, Pina JE, Rueff J, Gaspar JF (2010). Breast cancer risk and common single nucleotide polymorphisms in homologous recombination dna repair pathway genes *xrcc2*, *xrcc3*, *nbs1* and *rad51*. Cancer Epidemiol.

[21] Yu KD, Chen AX, Qiu LX, Fan L, Yang C, Shao ZM (2010). *Xrcc2* arg188his polymorphism is not directly associated with breast cancer risk: evidence from 37,369 subjects. Breast Cancer Res Treat.

[22] Pelttari LM, Kiiski JI, Ranta S, Vilske S, Blomqvist C, Aittomäki K, Nevanlinna H (2015). *Rad51*, *xrcc3*, and *xrcc2* mutation screening in finnish breast cancer families. Springerplus.

[23] Pooley KA, Baynes C, Driver KE, Tyrer J, Azzato EM, Pharoah PDP, Easton DF, Ponder BAJ, Dunning AM (2008). Common single-nucleotide polymorphisms in dna double-strand break repair genes and breast cancer risk. Cancer Epidemiol Biomark Prev.

[24] Lee KM, Choi JY, Kang C, Kang CP, Park SK, Cho H, Cho DY, Yoo KY, Noh DY, Ahn SH, Park C-G, Wei Q, Kang D (2005). Genetic polymorphisms of selected dna repair genes, estrogen and progesterone receptor status, and breast cancer risk. Clin Cancer Res.

[25] Webb PM, Hopper JL, Newman B, Chen X, Kelemen L, Giles GG, Southey MC, Chenevix-Trench G, Spurdle AB (2005). Double-strand break repair gene polymorphisms and risk of breast or ovarian cancer. Cancer Epidemiol Biomark Prev.

[26] Kuschel B, Auranen A, Mcbride S, Novik KL, Antoniou A, Lipscombe JM, Day NE, Easton DF, Ponder BAJ, Pharoah PDP, Dunning A (2002). Variants in dna double-strand break repair genes and breast cancer susceptibility. Hum Mol Genet.

[27] García-Closas M, Egan KM, Newcomb PA, Brinton LA, Titus-Ernstoff L, Chanock S, Welch R, Lissowska J, Peplonska B, Szeszenia-Dabrowska N, Zatonski W, Bardin-Mikolajczak A, Struewing JP (2006). Polymorphisms in dna double-strand break repair genes and risk of breast cancer: two population-based studies in usa and poland, and meta-analyses. Hum Genet.

[28] Kong B, Lv ZD, Chen L, Shen RW, Jin LY, Yang ZC (2015). Lack of an association between *xrcc2* r188h polymorphisms and breast cancer: an update meta-analysis involving 35,422 subjects. Int J Clin Exp Med.

[29] Xu K, Song X, Chen Z, Qin C, He Y (2014). *Xrcc2* rs3218536 polymorphism decreases the sensitivity of colorectal cancer cells to poly (adp-ribose) polymerase 1 inhibitor. Oncol Lett.

[30] Hilbers FS, Luijsterburg MS, Wiegant WW, Meijers CM, Völker-Albert M, Boonen RA, Van Asperen CJ, Devilee P, Van Attikum H (2016). Functional analysis of missense variants in the putative breast cancer susceptibility gene *xrcc2*. Hum Mutat.

[31] Brooks J, Shore RE, Zeleniuch-Jacquotte A, Currie D, Afanasyeva Y, Koenig KL, Arslan AA, Toniolo P, Wirgin I (2008). Polymorphisms in *rad51*, *xrcc*2, and *xrcc3* are not related to breast cancer risk. Cancer Epidemiol Biomark Prev.

[32] Rafii S, O’Regan P, Xinarianos G, Azmy I, Stephenson T, Reed M, Meuth M, Thacker J, Cox A (2002). A potential role for the *xrcc2* r188h polymorphic site in dna-damage repair and breast cancer. Hum Mol Genet.

[33] Jiao L, Hassan MM, Bondy ML, Wolff RA, Evans DB, Abbruzzese JL, Li D (2008). *Xrcc2* and *xrcc3* gene polymorphism and risk of pancreatic cancer. Am J Gastroenterol.

[34] Jakubowska A, Rozkrut D, Antoniou A, Hamann U, Scott RJ, Mcguffog L, Healy S, Sinilnikova OM, Rennert G, Lejbkowicz F, Flugelman A, Andrulis IL, Glendon G, Ozcelik H, Thomassen M, Paligo M, Aretini P, Kantala J, Aroer B, Von Wachenfeldt A, Liljegren A, Loman N, Herbst K, Kristoffersson U, Rosenquist R, Karlsson P, Stenmark-Askmalm M, Melin B, OCGN, SWE-BRCA (2012). Association of *phb* 1630 c>t and *mthfr* 677 c>t polymorphisms with breast and ovarian cancer risk in *brca1/2* mutation carriers: results from a multicenter study. Br J Cancer.

[35] Leal MF, Cirilo PD, Mazzotti TK, Calcagno DQ, Wisnieski F, Demachki S, Martinez MC, Assumpção PP, Chammas R, Burbano RR, Smith MC (2014). Prohibitin expression deregulation in gastric cancer is associated with the 3′ untranslated region 1630 c>t polymorphism and copy number variation. PLoS One.

[36] Manjeshwar S, Branam DE, Lerner MR, Brackett DJ, Jupe ER, Tumor suppression by the prohibitin gene 3’untranslated region rna in human breast cancer. Cancer Res. 2003;63:5251–6. 14500355

[37] Webster LR, Provan PJ, Graham DJ, Byth K, Walker RL, Davis S, Salisbury EL, Morey AL, Ward RL, Hawkins NJ, Clarke CL, Meltzer PS, Balleine RL (2013). Prohibitin expression is associated with high grade breast cancer but is not a driver of amplification at 17q21.33. Pathology.

[38] Najm MZ, Akhtar MS, Ahmad I, Sadaf S, Mallick MN, Kausar MA, Chattopadhyay S, Ahad A, Zaidi S, Husain SA, Siddiqui WA (2012). Mutational analysis of prohibitin - a highly conserved gene in indian female breast cancer cases. Asian Pac J Cancer Prev.

[39] Jakubowska A, Jaworska K, Cybulski C, Janicka A, Szymańska-Pasternak J, Lener M, Narod SA, Lubiński J, IHCC-Breast Cancer Study Group (2009). Do *brca1* modifiers also affect the risk of breast cancer in non-carriers?. Eur J Cancer.

[40] Jupe ER, Badgett AA, Neas BR, Craft MA, Mitchell DS, Resta R, Mulvihill JJ, Aston CE, Thompson LF (2001). Single nucleotide polymorphism in prohibitin 3’untranslated region and breast-cancer susceptibility. Lancet.

[41] Karakus N, Kara N, Ulusoy AN (2008). Lack of association between prohibitin 3’untranslated region c>t polymorphism and breast cancer in a turkish population. DNA Cell Biol.

[42] Spurdle AB, Hopper JL, Chen X, MRE M, Giles GG, Newman B, Chenevix-Trench G (2002). Prohibitin 3’untranslated region polymorphism and breast cancer risk in australian women. Lancet.

[43] Beeghly-Fadiel A, Lu W, Gao Y-T, Long J, Deming SL, Cai Q, Zheng Y, Shu X, Zheng W (2010). E-cadherin polymorphisms and breast cancer susceptibility: a report from the shanghai breast cancer study. Breast Cancer Res Treat.

[44] Zhao L, Gu A, Ji G, Zou P, Zhao P, Lu A (2012). The association between *atm* ivs 22–77 t>c and cancer risk: a meta-analysis. PLoS One.

[45] Mehdipour P, Mahdavi M, Mohammadi-Asl J, Atri M (2011). Importance of *atm* gene as a susceptible trait: predisposition role of d1853n polymorphism in breast cancer. Med Oncol.

[46] Schrauder M, Frank S, Strissel PL, Lux MP, Bani MR, Rauh C, Sieber CC, Heusinger K, Hartmann A, Schulz-Wendtland R, Strick R, Beckmann MW, Fasching PA (2008). Single nucleotide polymorphism d1853n of the *atm* gene may alter the risk for breast cancer. J Cancer Res Clin Oncol.

[47] Gao LB, Xm P, Sun H, Wang X, Rao L, Li LJ, Liang WB, Lv ML, Yang WZ, Zhang L (2010). The association between *atm* d1853n polymorphism and breast cancer susceptibility: a meta-analysis. J Exp Clin Cancer Res.

[48] Concannon P, Haile RW, Børresen-Dale AL, Rosenstein BS, Gatti RA, Teraoka SN, Diep TA, Jansen L, Atencio DP, Langholz B, Capanu M, Liang X, Begg CB, Thomas DC, Bernstein L, Olsen JH, Malone KE, Lynch CF, Anton-Culver H, Bernstein JL (2008). Variants in the *atm* gene associated with a reduced risk of contralateral breast cancer. Cancer Res.

