# Supplementary material for: Genetic determinants of sporadic breast cancer in Sri Lankan women
Source: BMC Cancer. 2018 Feb 13;18:180. doi: 10.1186/s12885-018-4112-4 (PMC5809862; doi:10.1186/s12885-018-4112-4)
Supplement: Supplementary file 1 — Table S1. List of haplotype-tagging single nucleotide polymorphisms selected for genotyping. Table S1 shows the list of haplotype-tagging single nucleotide polymorphisms which were selected for genotyping including detailed information about the genes, chromosomes, locations and putative functional scores of the genetic variants. (DOCX 23 kb) [file 12885_2018_4112_MOESM1_ESM.docx]

**Table S1: List of haplotype-tagging single nucleotide polymorphisms selected for genotyping**

| **S/No.** | **Single nucleotide polymorphism** | **Gene** | **Chromosome** | **Location** | **Functional score*** |
| --- | --- | --- | --- | --- | --- |
| 1 | rs7193955 | ***ABCC12*** | 16 | Exonic | 0.599 |
| 2 | rs1130214 | ***AKT1*** | 14 | 5ꞌUTR | 0-.176 |
| 3 | rs3733961 | ***APC*** | 5 | 3ꞌUTR | 0.268 |
| 4 | rs459552 | ***APC*** | 5 | Exonic | 0.844 |
| 5 | rs1801516 | ***ATM*** | 11 | Exonic | 0.425 |
| 6 | rs3092836 | ***ATM*** | 11 | 3ꞌUTR | 0.176 |
| 7 | rs4585 | ***ATM*** | 11 | 3ꞌUTR | 0.1 |
| 8 | rs799917 | ***BRCA1*** | 17 | Exonic | 0.518 |
| 9 | rs1799944 | ***BRCA2*** | 13 | Exonic | 0.368 |
| 10 | rs144848 | ***BRCA2*** | 13 | Exonic | 0.266 |
| 11 | rs15869 | ***BRCA2*** | 13 | 3ꞌUTR | 0.208 |
| 12 | rs11571836 | ***BRCA2*** | 13 | 3ꞌUTR | 0.176 |
| 13 | rs17860433 | ***CASP8*** | 2 | 3ꞌUTR | 0.176 |
| 14 | rs7177 | ***CCND1*** | 11 | 3ꞌUTR | 0.208 |
| 15 | rs13689 | ***CDH1*** | 16 | 3ꞌUTR | 0.176 |
| 16 | rs34330 | ***CDKN1B*** | 12 | 5ꞌUTR | 0.268 |
| 17 | rs2066827 | ***CDKN1B*** | 12 | Exonic | 1.0 |
| 18 | rs7330 | ***CDKN1B*** | 12 | 3ꞌUTR | 0.208 |
| 19 | rs3088440 | ***CDKN2A*** | 9 | 3ꞌUTR | 0.176 |
| 20 | rs20551 | ***EP300*** | 22 | Exonic | 0.103 |
| 21 | rs4252661 | ***ERBB2*** | 17 | 3ꞌUTR | 0.22 |
| 22 | rs4647414 | ***FANCC*** | 9 | 5ꞌUTR | 0.208 |
| 23 | rs4647558 | ***FANCC*** | 9 | 3ꞌUTR | 0.176 |
| 24 | rs3740615 | ***FANCF*** | 11 | 5ꞌUTR | 0.101 |
| 25 | rs13317 | ***FGFR1*** | 8 | 3ꞌUTR | 0.573 |
| 26 | rs1047111 | ***FGFR2*** | 10 | 5ꞌUTR | 0.101 |
| 27 | rs7144658 | ***FOXA1*** | 14 | Exonic | 0.103 |
| 28 | rs9746 | ***GATA3*** | 10 | 3ꞌUTR | 0.213 |
| 29 | rs1058240 | ***GATA3*** | 10 | 3ꞌUTR | 0.208 |
| 30 | rs2229360 | ***GATA3*** | 10 | 3ꞌUTR | 0.208 |
| 31 | rs299290 | ***HMMR*** | 5 | Exonic | 0.237 |
| 32 | rs712 | ***KRAS*** | 12 | 3ꞌUTR | 0.191 |
| 33 | rs12416967 | ***LSP1*** | 11 | 5upstream | 0.5 |
| 34 | rs907613 | ***LSP1*** | 11 | 5ꞌUTR | 0.5 |
| 35 | rs2870820 | ***MDM2*** | 12 | 5upstream | 0.268 |
| 36 | rs1799977 | ***MLH1*** | 3 | Exonic | 0.273 |
| 37 | rs2269529 | ***MYH9*** | 22 | Exonic | 0.5 |
| 38 | rs2481 | ***MYH9*** | 22 | 3ꞌUTR | - |
| 39 | rs9995 | ***NBN*** | 8 | 3ꞌUTR | 0.208 |
| 40 | rs14448 | ***NBN*** | 8 | 3ꞌUTR | 0.176 |
| 41 | rs1805794 | ***NBN*** | 8 | Exonic | 0.899 |
| 42 | rs2071002 | ***NQO2*** | 6 | 5ꞌUTR | 0.242 |
| 43 | rs17136117 | ***NQO2*** | 6 | Exonic | 0.869 |
| 44 | rs1143684 | ***NQO2*** | 6 | Exonic | 0.407 |
| 45 | rs17300141 | ***NQO2*** | 6 | Exonic | 0.631 |
| 46 | rs2228006 | ***PMS2*** | 7 | Exonic | 0.5 |
| 47 | rs701848 | ***PTEN*** | 10 | 3ꞌUTR | 0.084 |
| 48 | rs6917 | ***PHB*** | 17 | 3ꞌUTR | 0.95 |
| 49 | rs1049620 | ***PHB*** | 17 | 3ꞌUTR | 0.623 |
| 50 | rs17337252 | ***RB1CC1*** | 8 | Exonic | 0.117 |
| 51 | rs3741378 | ***SIPA1*** | 11 | Exonic | 0.322 |
| 52 | rs2234167 | ***TNFRSF14*** | 1 | Exonic | 0.365 |
| 53 | rs2909430 | ***TP53*** | 17 | 5ꞌUTR | 0.1 |
| 54 | rs25489 | ***XRCC1*** | 19 | Exonic | 0.309 |
| 55 | rs25487 | ***XRCC1*** | 19 | Exonic | 0.407 |
| 56 | rs3218536 | ***XRCC2*** | 7 | Exonic | 0.195 |
| 57 | rs3218552 | ***XRCC2*** | 7 | 3ꞌUTR | 0.176 |
| 58 | rs3218550 | ***XRCC2*** | 7 | 3ꞌUTR | 0.208 |

*Based on SNPnexus: Abu Z Dayem Ullah, Nicholas R Lemoine and Claude Chelala, A practical guide for the functional annotation of genetic variations using SNPnexus, Briefings in Bioinformatics, 2013, 14(4):437-47.
