# Supplementary material for: Genetic determinants of sporadic breast cancer in Sri Lankan women
Source: BMC Cancer. 2018 Feb 13;18:180. doi: 10.1186/s12885-018-4112-4 (PMC5809862; doi:10.1186/s12885-018-4112-4)
Supplement: Supplementary file 2 — Table S2 Relationship between the genotyped single nucleotide polymorphisms and breast cancer risk. Table S2 shows the list of haplotype-tagging single nucleotide polymorphisms which were genotyped in the study cohort and their association with breast cancer risk. (DOCX 19 kb) [file 12885_2018_4112_MOESM2_ESM.docx]

**Table S2: Relationship between the genotyped single nucleotide polymorphisms and breast cancer risk**

| **Chromosome** | **Gene** | **Single nucleotide polymorphism** | **Location** | **Variant allele** | **Variant allele frequency [Cases]** | **Variant allele frequency**  **[Controls]** | **Odds ratio** | **95% confidence interval** | | ***P*-value** | **Association with breast cancer** | |  |
| --- | --- | --- | --- | --- | --- | --- | --- | --- | --- | --- | --- | --- | --- |
| 1 | *TNFRSF14* | rs2234167 | EXONIC | A | 0.1275 | 0.1121 | 1.163 | 0.837 - 1.615 | | 0.3686 | NIL | |  |
| 2 | *CASP8* | rs17860433 | 3ꞌUTR | G | 0.05587 | 0.04885 | 1.147 | 0.7204 - 1.827 | | 0.5626 | NIL | |  |
| 3 | *MLH1* | rs1799977 | EXONIC | G | 0.1189 | 0.1149 | 1.038 | 0.7514 - 1.435 | | 0.8195 | NIL | |  |
| 5 | *APC* | rs459552 | EXONIC | T | 0.1977 | 0.2083 | 0.9382 | 0.7252 - 1.214 | | 0.6269 | NIL | |  |
| 5 | *APC* | rs3733961 | 3ꞌUTR | T | 0.09026 | 0.09626 | 0.929 | 0.6432 - 1.342 | | 0.6947 | NIL | |  |
| 6 | *NQO2* | rs2071002 | 5ꞌUTR | C | 0.2636 | 0.2701 | 0.9657 | 0.757 - 1.232 | | 0.7784 | NIL | |  |
| 6 | *NQO2* | rs17136117 | EXONIC | G | 0.08739 | 0.06897 | 1.295 | 0.8721 - 1.923 | | 0.2001 | NIL | |  |
| 6 | *NQO2* | rs1143684 | EXONIC | C | 0.2235 | 0.2399 | 0.9147 | 0.7162 - 1.168 | | 0.475 | NIL | |  |
| 6 | *NQO2* | rs17300141 | EXONIC | A | 0.04441 | 0.04023 | 1.102 | 0.6639 - 1.83 | | 0.7068 | NIL | |  |
| 7 | *PMS2* | rs2228006 | EXONIC | T | 0.09026 | 0.08764 | 1.034 | 0.7092 - 1.508 | | 0.8608 | NIL | |  |
| 7 | *XRCC2* | rs3218552 | 3ꞌUTR | T | 0.1218 | 0.1121 | 1.093 | 0.7955 - 1.501 | | 0.5844 | NIL | |  |
| 7 | *XRCC2* | rs3218550 | 3ꞌUTR | T | 0.1517 | 0.1049 | 1.525 | 1.107 - 2.101 | | 0.009837 | INCREASED RISK | |  |
| 7 | *XRCC2* | rs3218536 | EXONIC | T | 0.1074 | 0.1135 | 0.9406 | 0.6736 - 1.313 | | 0.7194 | NIL | |  |
| 8 | *FGFR1* | rs13317 | 3ꞌUTR | C | 0.1232 | 0.1178 | 1.054 | 0.7596 - 1.462 | | 0.7537 | NIL | |  |
| 8 | *RB1CC1* | rs17337252 | EXONIC | G | 0.4799 | 0.5043 | 0.911 | 0.7418 - 1.119 | | 0.3741 | NIL | |  |
| 8 | *NBN* | rs14448 | 3ꞌUTR | G | 0.2364 | 0.2328 | 1.021 | 0.7956 - 1.309 | | 0.8722 | NIL | |  |
| 8 | *NBN* | rs9995 | 3ꞌUTR | G | 0.2951 | 0.3175 | 0.9015 | 0.7191 - 1.13 | | 0.3687 | NIL | |  |
| 8 | *NBN* | rs1805794 | EXONIC | G | 0.4456 | 0.4655 | 0.9241 | 0.7498 - 1.139 | | 0.4588 | NIL | |  |
| 9 | *CDKN2A* | rs3088440 | 3ꞌUTR | A | 0.2178 | 0.2213 | 0.9788 | 0.7548 - 1.269 | | 0.8716 | NIL | |  |
| 9 | *FANCC* | rs4647558 | 3ꞌUTR | T | 0.4527 | 0.4741 | 0.918 | 0.7442 - 1.132 | | 0.4246 | NIL | |  |
| 9 | *FANCC* | rs4647414 | 5ꞌUTR | G | 0.06447 | 0.04741 | 1.387 | 0.8723 - 2.206 | | 0.1667 | NIL | |  |
| 10 | *GATA3* | rs2229360 | 3ꞌUTR | T,. | 0.09456 | 0.0819 | 1.172 | 0.8075 - 1.702 | | 0.4034 | NIL | |  |
| 10 | *GATA3* | rs9746 | 3ꞌUTR | G | 0.1848 | 0.1983 | 0.914 | 0.6966 - 1.199 | | 0.5162 | NIL | |  |
| 10 | *GATA3* | rs1058240 | 3ꞌUTR | G | 0.07736 | 0.07759 | 0.997 | 0.6779 - 1.466 | | 0.9878 | NIL | |  |
| 10 | *PTEN* | rs701848 | 3ꞌUTR | T | 0.3181 | 0.3463 | 0.8923 | 0.7223 - 1.102 | | 0.2903 | NIL | |  |
| 11 | *LSP1* | rs907613 | 5ꞌUTR | A | 0.2479 | 0.2385 | 1.05 | 0.8258 - 1.336 | | 0.6897 | NIL | |  |
| 11 | *LSP1* | rs12416967 | 5UPSTREAM | G | 0.1433 | 0.171 | 0.8102 | 0.606 - 1.083 | | 0.1554 | NIL | |  |
| 11 | *FANCF* | rs3740615 | 5ꞌUTR | A | 0.04155 | 0.04167 | 0.997 | 0.5882 | 1.69 - 0.9911 | | | NIL | |
| 11 | *SIPA1* | rs3741378 | EXONIC | T | 0.08883 | 0.09626 | 0.9152 | 0.6369 | 1.315 - 0.632 | | | NIL | |
| 11 | *CCND1* | rs7177 | 3ꞌUTR | C | 0.5 | 0.4497 | 1.233 | 0.9912 | 1.535 - 0.05999 | | | NIL | |
| 11 | *ATM* | rs1801516 | EXONIC | A | 0.05444 | 0.08764 | 0.5948 | 0.3894 | 0.9088 - 0.01629 | | | PROTECTIVE | |
| 11 | *ATM* | rs3092836 | 3ꞌUTR | C | 0.05874 | 0.06609 | 0.8877 | 0.5814 | 1.355 - 0.5811 | | | NIL | |
| 11 | *ATM* | rs4585 | 3ꞌUTR | G | 0.3424 | 0.3247 | 1.082 | 0.8668 | 1.351 - 0.4857 | | | NIL | |
| 12 | *CDKN1B* | rs34330 | 5ꞌUTR | T | 0.2292 | 0.2428 | 0.9221 | 0.7134 | 1.192 - 0.5356 | | | NIL | |
| 12 | *CDKN1B* | rs2066827 | EXONIC | G | 0.3204 | 0.3376 | 0.9217 | 0.7334 | 1.158 - 0.4846 | | | NIL | |
| 12 | *CDKN1B* | rs7330 | 3ꞌUTR | C | 0.2923 | 0.296 | 0.9816 | 0.7762 | 1.241 - 0.8768 | | | NIL | |
| 12 | *KRAS* | rs712 | 3ꞌUTR | A | 0.3138 | 0.2816 | 1.163 | 0.9258 | 1.46 - 0.1947 | | | NIL | |
| 12 | *MDM2* | rs2870820 | 5UPSTREAM | T | 0.2135 | 0.2291 | 0.9165 | 0.715 | 1.175 - 0.4911 | | | NIL | |
| 13 | *BRCA2* | rs144848 | EXONIC | C | 0.3868 | 0.3793 | 1.032 | 0.832 | 1.28 - 0.7736 | | | NIL | |
| 13 | *BRCA2* | rs1799944 | EXONIC | G | 0.1189 | 0.1121 | 1.069 | 0.7705 | 1.482 - 0.6909 | | | NIL | |
| 13 | *BRCA2* | rs15869 | 3ꞌUTR | C | 0.2307 | 0.2328 | 0.9891 | 0.7783 | 1.257 - 0.9287 | | | NIL | |
| 13 | *BRCA2* | rs11571836 | 3ꞌUTR | G | 0.1825 | 0.1758 | 1.046 | 0.7967 | 1.373 - 0.7475 | | | NIL | |
| 14 | *FOXA1* | rs7144658 | EXONIC | C | 0.4282 | 0.4609 | 0.8738 | 0.7053 | 1.082 - 0.2169 | | | NIL | |
| 14 | *AKT1* | rs1130214 | 5ꞌUTR | A | 0.1351 | 0.1566 | 0.8534 | 0.6414 | 1.136 - 0.2769 | | | NIL | |
| 16 | *ABCC12* | rs7193955 | EXONIC | G | 0.1977 | 0.2126 | 0.9177 | 0.7134 | 1.181 - 0.5041 | | | NIL | |
| 16 | *CDH1* | rs13689 | 3ꞌUTR | C | 0.09885 | 0.1351 | 0.6965 | 0.4982 | 0.9737 - 0.03437 | | | PROTECTIVE | |
| 17 | *TP53* | rs2909430 | 5ꞌUTR | C | 0.1619 | 0.181 | 0.8778 | 0.6672 | 1.155 - 0.3518 | | | NIL | |
| 17 | *ERBB2* | rs4252661 | 3ꞌUTR | C | 0.1017 | 0.09052 | 1.139 | 0.796 | 1.63 - 0.4767 | | | NIL | |
| 17 | *BRCA1* | rs799917 | EXONIC | G | 0.4612 | 0.4583 | 1.012 | 0.8179 | 1.252 - 0.9136 | | | NIL | |
| 17 | *PHB* | rs1049620 | 3ꞌUTR | T | 0.3252 | 0.3372 | 0.9489 | 0.7615 | 1.182 - 0.6405 | | | NIL | |
| 17 | *PHB* | rs6917 | 3ꞌUTR | A | 0.2937 | 0.2298 | 1.41 | 1.102 | 1.803 - 0.006227 | | | INCREASED RISK | |
| 19 | *XRCC1* | rs25487 | EXONIC | T | 0.3181 | 0.342 | 0.9017 | 0.7247 | 1.122 - 0.3537 | | | NIL | |
| 19 | *XRCC1* | rs25489 | EXONIC | T | 0.1433 | 0.1437 | 0.9966 | 0.7369 | 1.348 - 0.9824 | | | NIL | |
| 22 | *MYH9* | rs2481 | 3ꞌUTR | A | 0.4756 | 0.4986 | 0.9158 | 0.7455 | 1.125 - 0.4023 | | | NIL | |
| 22 | *MYH9* | rs2269529 | EXONIC | T | 0.457 | 0.4569 | 1 | 0.8188 | 1.222 - 0.9965 | | | NIL | |
| 22 | *EP300* | rs20551 | EXONIC | G | 0.3532 | 0.3732 | 0.9164 | 0.7345 | 1.143 - 0.4391 | | | NIL | |
